# The Biological Conservative Reconstruction (BIOCORN) Procedure: Redefining Surgical Treatment of Gastroesophageal Reflux Disease (GERD) and Hiatal Hernia Through Anatomical Preservation

**DOI:** 10.7759/cureus.94865

**Published:** 2025-10-18

**Authors:** Mark Salib, John Salib, Matthew Phillips

**Affiliations:** 1 School of Medicine, St. George's University School of Medicine, St. George's, GRD; 2 General Surgery, Community First Medical Center, Chicago, USA

**Keywords:** anatomical restoration, anti-reflux surgery, bicorn procedure, fundoplication alternative, gastroesophageal reflux, hiatal hernia, minimally invasive surgery, physiological function preservation, surgical innovation

## Abstract

The Biological Conservative Reconstruction (BIOCORN) procedure is a laparoscopic, anatomically conservative approach for managing gastroesophageal reflux disease and hiatal hernia. Unlike traditional wrap-based surgeries, BICORN restores the native gastroesophageal junction and angle of His without creating a restrictive wrap, preserving physiological functions such as belching and vomiting. This narrative review synthesized evidence from multiple studies identified through a comprehensive literature search using systematic selection and independent, blinded review to minimize bias. Clinical data indicate that BICORN provides high patient satisfaction, durable symptom relief, and effective reflux control while minimizing common postoperative complications, including dysphagia, gas-bloat syndrome, and impaired gastric emptying. Reported hernia recurrence rates vary, emphasizing the importance of careful patient selection and meticulous surgical technique. Postoperative assessments suggest preserved esophageal physiology despite minimal changes in lower esophageal sphincter pressures, supporting the procedure's anatomical and functional approach. Despite these positive outcomes, gaps remain in long-term efficacy data, direct comparisons with traditional antireflux techniques, and criteria for optimal patient selection. Future research should focus on controlled studies, long-term outcomes, and patient-reported measures to define BICORN's role as a physiologically conservative surgical option. This review highlights BICORN as a promising approach for patients seeking effective reflux management while maintaining natural anatomy and function.

## Introduction and background

Gastroesophageal reflux disease (GERD) affects up to 20% of adults in Western countries. It frequently coexists with hiatal hernia, a condition in which part of the stomach herniates through the diaphragm into the thoracic cavity [1,2]. While many patients achieve symptom control through lifestyle modifications and pharmacologic therapy, particularly proton pump inhibitors, about 40% of patients remain symptomatic or develop complications such as esophagitis or Barrett's esophagus [2]. Surgical correction of the underlying anatomical and functional abnormalities is necessary for these individuals [1,3].

The current gold standard for surgical management of GERD and hiatal hernia is fundoplication, most commonly the Nissen procedure, which reinforces the lower esophageal sphincter (LES) by wrapping the gastric fundus around the distal esophagus [4]. Although highly effective in reducing reflux, this approach is associated with notable postoperative side effects, including dysphagia, gas-bloat syndrome, and an inability to belch or vomit, which significantly affect patient satisfaction and quality of life [2]. Patients with large paraesophageal hernias or esophageal motility disorders may be suboptimal candidates for this technique [5,6].

The Biological Conservative Reconstruction (BICORN) procedure was first theorized in 2000 as a response to the limitations of traditional fundoplication techniques. It was subsequently performed for the first time in 2004 and later refined in 2014. BICORN offers an anatomically conservative alternative to fundoplication that aims to restore the native gastroesophageal junction (GEJ) and the angle of His without creating a wrap [7,8]. Unlike traditional antireflux surgeries, BICORN preserves the physiological ability to belch and vomit, thereby reducing fundoplication-related side effects. The laparoscopic approach involves repositioning the stomach into the abdominal cavity, repairing the hiatal defect typically without mesh, and securing the esophagus to the diaphragmatic crura with non-absorbable sutures [7].

Historically, the evolution of the BICORN technique reflected a paradigm shift in antireflux surgery. Since its introduction, the Nissen fundoplication has dominated surgical treatment for GERD but frequently altered the natural physiology of the GEJ, leading surgeons to question whether such modification was necessary for effective reflux control [4,5]. The BICORN approach emerged from this reconsideration, prioritizing restoration over reconstruction. Early European adopters, particularly in Germany and Switzerland, applied the technique in patients for whom fundoplication was either contraindicated or failed [7]. Intermediate-term follow-up experiences demonstrated excellent symptom relief, preservation of physiological function, and high patient satisfaction rates, with up to 91% of patients reporting outstanding outcomes and over 90% discontinuing proton pump inhibitors postoperatively, although hernia recurrence rates of 10% to 30% over 5 to 10 years have been reported [9-12].

This narrative review synthesizes current evidence on the BICORN procedure, outlining its anatomical basis, surgical technique, indications, contraindications, complications, and clinical outcomes. By contextualizing BICORN within the broader evolution of antireflux surgery, this paper aims to clarify its role as a physiologically preservative and anatomically restorative option for managing GERD and hiatal hernia.

## Review

Methods

**Figure 1:**
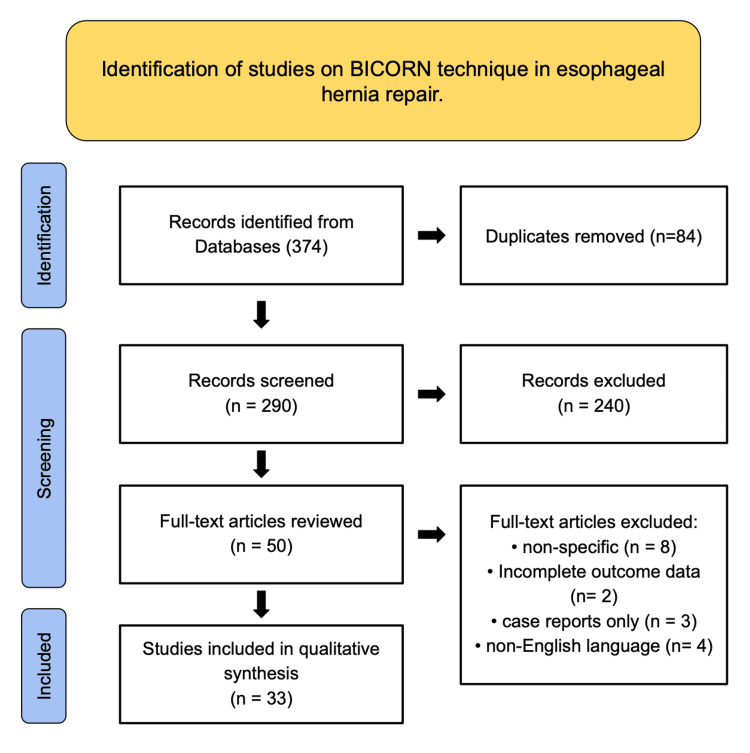
PRISMA Flow Diagram Showing Study Selection for the BICORN Technique in Esophageal Hernia Repair. Records were identified, duplicates removed, and titles and abstracts screened. Full-text articles were reviewed, with exclusions for non-specific focus, incomplete data, case report format, and non-English language. Studies meeting the inclusion criteria were analyzed. PRISMA: Preferred Reporting Items for Systematic Reviews and Meta-Analyses; BIOCORN: Biological Conservative Reconstruction

A comprehensive qualitative literature search was conducted in accordance with the Preferred Reporting Items for Systematic Reviews and Meta-Analyses (PRISMA) 2020 guidelines (as depicted in Figure 1) to identify studies evaluating the BICORN technique in esophageal and hiatal hernia repair. Searches were performed across three major electronic databases: PubMed, Embase, and Scopus, covering publications from January 2000 through July 2025. The search strategy employed a combination of Medical Subject Headings (MeSH) and free-text terms, including but not limited to "BICORN," "hiatal hernia," "esophageal hernia repair," "laparoscopic technique," "antireflux surgery," and "biological reconstruction." Boolean operators such as "AND" and "OR" were applied to optimize sensitivity and specificity, ensuring the inclusion of all relevant literature.

A total of 374 records were initially identified across all databases. Following the removal of 84 duplicates, 290 unique records were screened by title and abstract. Studies were excluded if they lacked surgical detail, employed unrelated methodologies, or focused on non-BICORN techniques. The remaining 50 full-text articles were assessed for eligibility, with 17 subsequently excluded for reasons such as non-BICORN-specific techniques (n = 8), incomplete or missing outcome data (n = 2), single case reports only (n = 3), or non-English language publications (n = 4). Ultimately, 33 studies met the inclusion criteria and were incorporated into the qualitative synthesis, as summarized in Table 1.

**Table 1:** Summary of Key Literature on GERD Management and the BICORN Technique. Overview of 33 key publications included in the qualitative synthesis of this review. Studies span a range of publication types, including guidelines, systematic reviews, original research, and clinical trials, and cover topics such as GERD diagnosis and management, antireflux surgical approaches, the evolution of the BICORN technique, and long-term surgical outcomes. The table outlines each citation’s title and year, population or study type, classification, and a concise summary of the paper’s contributions. GERD: gastroesophageal reflux disease; BICORN: Biological Conservative Reconstruction

Cited Title & Year	Population/Number of Studies Reviewed	Type of Paper
Harsányi L, et al., Five-year clinical outcomes of RefluxStop surgery, 2025 [1]	50 patients (44 with 5-year follow-up)	Prospective Multicenter Trial
Eckhardt D, et al., HiatoGast 1 trial, 2019 [2]	~210 participants	Exploratory Semiblinded Trial
Sudarshan M & Raja S, Re-operative surgery after paraesophageal hernia repair, 2022 [3]	Literature review	Narrative Review
Chrysos E, et al., Laparoscopic vs open Nissen, 2002 [4]	102 patients undergoing Nissen fundoplication	Comparative Study
Frazzoni M, et al., Laparoscopic fundoplication, 2014 [5]	1,232 patients undergoing laparoscopic fundoplication	Observational/Cohort Study
Stefanidis D, et al., GERD surgery guidelines, 2010 [6]	Surgeons and patients undergoing anti-reflux surgery; literature review	Clinical Practice Guideline
Singhal VK, et al., Hiatal hernia trends, 2024 [7]	Literature review covering 10 years of hiatal hernia management studies	Literature Review
Jobe BA, et al., Transoral endoscopic fundoplication, 2008 [8]	Patients with GERD undergoing transoral endoscopic fundoplication	Original Research/Device Study
Yano F, et al., Laparoscopic hiatal hernia repair, 2021 [9]	Review of patients undergoing laparoscopic hiatal hernia repair	Review Article
Prassas D, et al., Long-term Nissen outcomes, 2017 [10]	108 patients followed long-term after laparoscopic Nissen fundoplication	Observational Cohort Study
Cadière GB, Anti-reflux surgery principles, 1994 [11]	General review; historical and clinical perspective	Review Article
Herregods TVK, et al., GERD pathophysiology, 2015 [12]	Adult patients with GERD; literature review	Review Article
Zhang R, et al., Angle of His reconstruction, 2022 [13]	Patients with GERD and hiatal hernia undergoing laparoscopic surgery	Original Research
Siemssen B, et al., PH4B-mesh hiatal repair, 2024 [14]	176 patients undergoing hiatal hernia repair without conventional fundoplication	Observational Study
Pandolfino JE, et al., Obesity & EGJ integrity, 2006 [15]	Adult patients; literature and clinical analysis	Review/Original Research
Slater BJ, et al., SAGES GERD surgery guidelines, 2021 [16]	Surgeons and GERD patients; guideline review	Clinical Practice Guideline
Oelschlager BK, et al., Biologic prosthesis hiatal hernia, 2006 [17]	Patients with large hiatal hernias	Randomized Controlled Trial
Patti MG, et al., Esophagus surgery anatomy & physiology, 1997 [18]	General surgical population; literature review	Review Article
Aslam N, et al., Minimally invasive endoscopic GERD therapies, 2023 [19]	Patients with GERD undergoing endoscopic treatment	Review/Literature Review
Mittal R, Vaezi MF, Esophageal motility disorders & GERD, 2020 [20]	Adult patients with motility disorders; literature review	Review Article
Wang R, et al., Global digestive disease burden, 2023 [21]	Systematic analysis of 204 countries from 1990–2019	Systematic Analysis
Sfara A, Dumitrascu DL, Hiatal hernia management update, 2019 [22]	Adult patients with hiatal hernia; literature review	Review Article
Katz PO, et al., GERD Diagnosis & management guidelines, 2013 [23]	Broad adult population with GERD; multiple guideline references	Clinical Guideline
Krause AJ, et al., Ambulatory reflux monitoring, 2024 [24]	Patients with chronic laryngeal symptoms undergoing reflux testing	Original Research
Fein M, et al., Ten-Year Laparoscopic antireflux outcomes, 2008 [25]	Patients undergoing laparoscopic antireflux surgery; 10-year follow-up	Observational Cohort Study
Tam V, et al., Mesh vs suture cruroplasty meta-analysis, 2016 [26]	Multiple studies comparing mesh and suture cruroplasty in large hiatal hernia repair	Systematic Review and Meta-Analysis
Lima DL, et al., Biosynthetic mesh hiatal repair review, 2023 [27]	Literature on hiatal hernia repair with biosynthetic mesh	Systematic Review
Louie BE, et al., Magnetic sphincter augmentation, 2019 [28]	Patients with GERD undergoing magnetic sphincter augmentation; one-year follow-up	Original Research/Device Study
Granderath FA, et al., Nissen with prosthetic hiatal closure, 2006 [29]	Patients undergoing laparoscopic Nissen fundoplication with prosthetic closure	Prospective Randomized Trial
Müller-Stich BP, et al., Mesh use in paraesophageal hernia, 2015 [30]	Multiple studies on mesh use in laparoscopic paraesophageal hernia repair	Meta-Analysis and Risk-Benefit Analysis
Kumar SS, et al., Hiatal hernia repair in geriatrics, 2024 [31]	Geriatric patients undergoing hiatal hernia repair across multiple centers	Multicenter Observational Study
Rosenthal R, et al., Long-term outcomes laparoscopic antireflux surgery, 2006 [32]	Patients undergoing laparoscopic antireflux surgery with long-term follow-up	Observational Cohort Study
Wu H, et al., Nissen vs Toupet vs LINX fundoplication, 2022 [33]	Patients with GERD undergoing different laparoscopic antireflux procedures	Comparative Study

Three independent reviewers were assigned to screen, extract, and synthesize data while blinded from each other to enhance the objectivity and methodological rigor of this review. Each reviewer independently compiled information into structured results sheets, extracting key study characteristics such as design, sample size, surgical approach, operative outcomes, recurrence rates, and postoperative complications. Upon completing individual data extraction, the reviewers compared findings and resolved discrepancies through consensus-based discussion, ensuring the synthesis reflected a balanced and evidence-driven interpretation of the literature.

Data were then qualitatively analyzed and synthesized into a narrative format to provide a cohesive overview of the anatomical principles, operative techniques, clinical outcomes, and complication profiles associated with the BICORN procedure. By employing blinded, independent data extraction, systematic PRISMA-based selection, and qualitative synthesis of heterogeneous evidence, this review aimed to minimize selection and interpretation bias while providing a comprehensive, high-quality overview of the current evidence base surrounding the BICORN procedure.

Surgical anatomy

**Figure 2:**
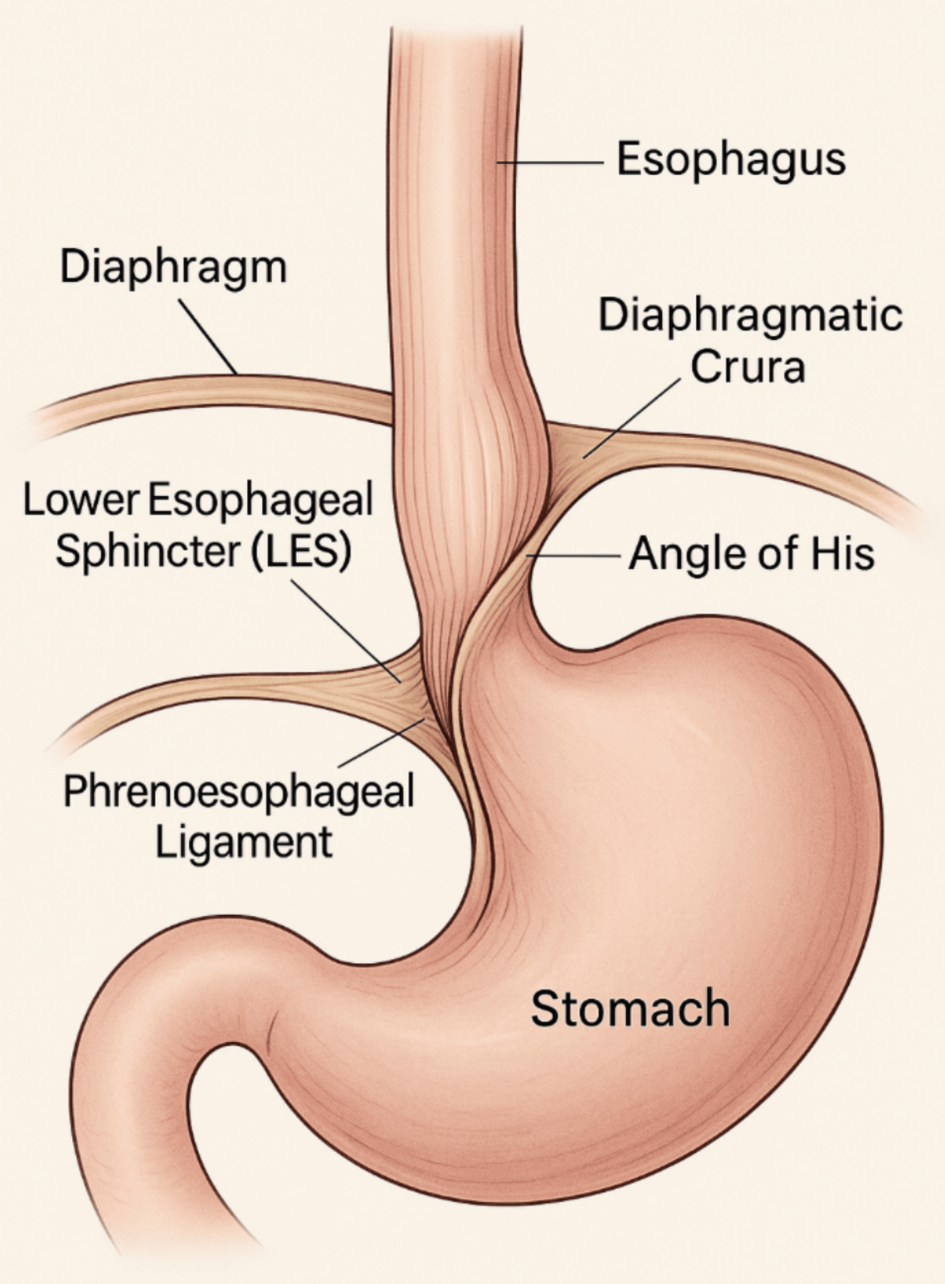
Key Anatomical Structures of the Gastroesophageal Junction (GEJ). The GEJ includes the LES, diaphragmatic crura, angle of His, and phrenoesophageal ligament, which together form a high-pressure barrier against reflux. The angle of His acts as a mechanical flap, while the phrenoesophageal ligament anchors the esophagus to the diaphragm. Disruption of these elements can lead to gastroesophageal reflux disease (GERD) or hiatal hernia. Image credit: Illustration designed and created by the authors.

The GEJ (as depicted in Figure 2) comprises the LES, diaphragmatic crura, angle of His, and phrenoesophageal ligament (structures that together maintain a high-pressure antireflux barrier) [13]. The angle of His acts as a mechanical flap valve, reinforcing LES competence. Disruption of this anatomy due to congenital defects, aging, increased intra-abdominal pressure, or esophageal shortening can weaken the barrier, leading to GERD or a hiatal hernia [14].

**Figure 3:**
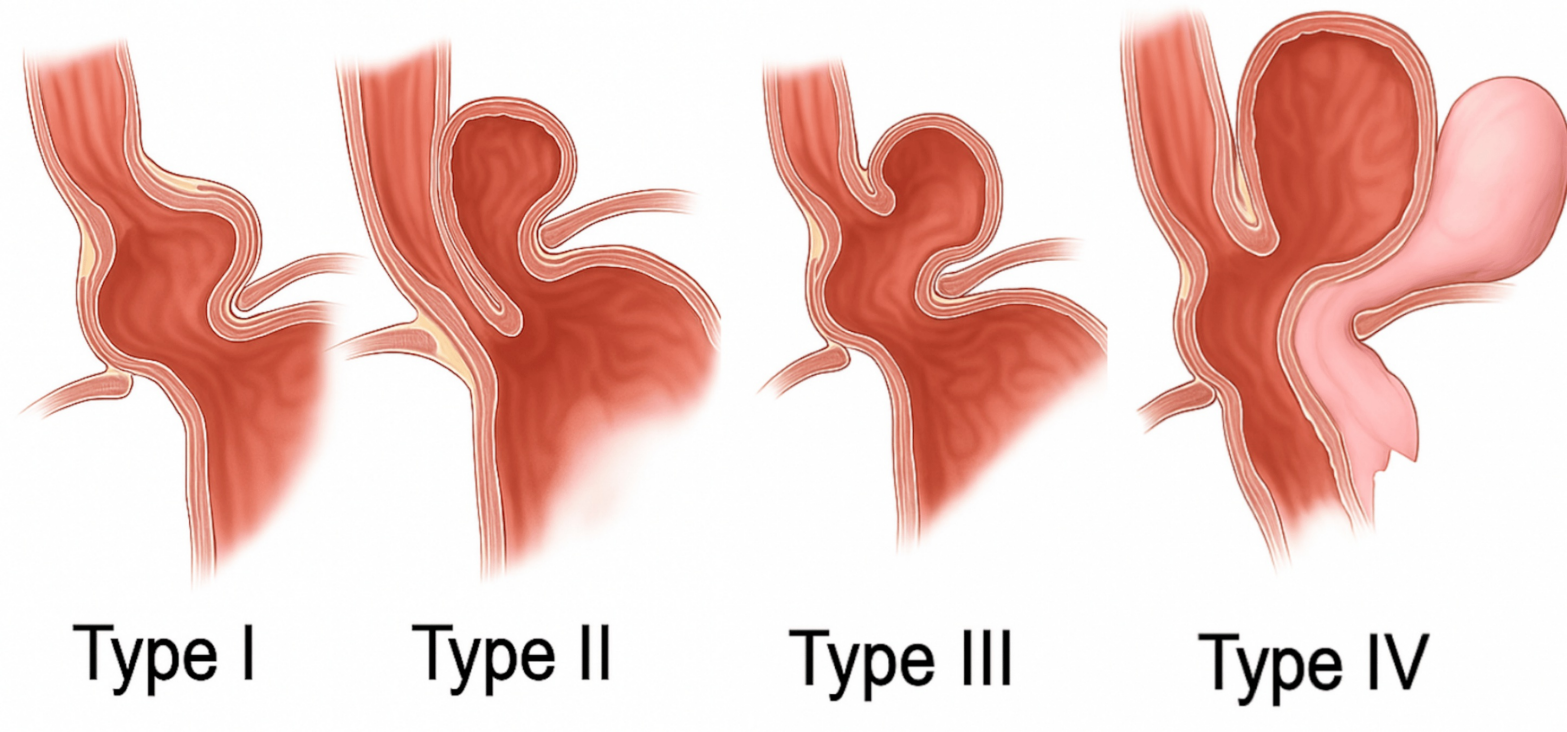
Hiatal Hernia Classifications. Hiatal hernias are categorized into four types based on the anatomical pattern of herniation through the esophageal hiatus. Type I (sliding) is the most common and involves upward displacement of the gastroesophageal junction. Type II (paraesophageal) features herniation of the gastric fundus alongside a normally positioned gastroesophageal junction. Type III combines the displacement of both the gastroesophageal junction and the fundus. Type IV is the most advanced form, with herniation of the stomach along with other abdominal organs. Image credit: Illustration designed and created by the authors.

Hiatal hernias are classified into four types (as depicted in Figure 3) based on the herniation pattern through the esophageal hiatus. Type I (sliding) is the most common (>90%), involving GEJ displacement into the thorax and often associated with GERD [13,14]. Type II (paraesophageal) occurs when the gastric fundus herniates adjacent to a usually positioned GEJ. Type III combines features of Types I and II [13,14]. Type IV is the most complex, involving herniation of other abdominal organs like the colon or spleen alongside the stomach [15].

Understanding these classifications is essential for surgical planning. While procedures like Nissen fundoplication aim to restore LES function, they may distort anatomy and cause complications. The BICORN procedure offers a more anatomically conservative approach, preserving native junctional relationships while effectively managing reflux and herniation.

Indications and contraindications

The BICORN procedure is best suited for patients with symptomatic GERD or hiatal hernia who have not responded well to medical therapy or who prefer to avoid the long-term side effects of medications like proton pump inhibitors. It is also a practical approach for preserving native anatomy and physiological function relative to traditional antireflux procedures. Unlike Nissen or Toupet fundoplication, which often involve wrapping part of the stomach around the esophagus, the BICORN procedure restores the normal junctional relationships without creating a tight mechanical barrier. This can help avoid common issues such as gas bloating, trouble swallowing, and difficulty belching, side effects that can significantly impact daily comfort and quality of life [16,17].

However, not all patients are good candidates. The procedure is not recommended for individuals with a significantly shortened esophagus, as they may require a more complex repair, such as a Collis gastroplasty. It is also not ideal for large or complicated paraesophageal hernias that might need mesh reinforcement. Patients with poor esophageal motility, as measured by manometry, may benefit from a partial fundoplication that considers their reduced ability to propel food down the esophagus [17]. Other important considerations include a history of upper GI surgery, which may make the anatomy too altered for this approach, or a prior diagnosis of gastric or esophageal cancer, where more extensive surgical planning is required [18-20].

Surgical technique

The BICORN procedure is a minimally invasive approach designed to anatomically restore the GEJ without using a fundoplication wrap. The goals are to reduce the herniated stomach, repair the hiatal defect, and re-establish the angle of His, thereby recreating the physiological antireflux barrier. Unlike traditional wrap-based techniques, BICORN maintains natural gastric venting mechanisms, preserving the ability to belch and vomit while effectively controlling reflux [7,8].

The patient is positioned supine with a reverse Trendelenburg tilt to promote gravitational retraction of abdominal contents and facilitate exposure of the hiatus [16]. Pneumoperitoneum is established, and five laparoscopic ports are inserted, typically four 5 mm and one 12 mm, strategically positioned to optimize visualization and ergonomic access to the hiatus.

Hiatal dissection begins with careful mobilization of the distal esophagus and proximal stomach. The phrenoesophageal ligament is divided circumferentially, and any adhesions between the esophagus, stomach, and diaphragm are lysed. This mobilization allows the surgeon to restore the natural relationship between the esophagus and crura. Both vagal trunks are preserved throughout dissection to prevent postoperative gastroparesis and maintain coordinated gastric motility [17].

After complete mobilization, the herniated stomach is reduced into the abdominal cavity. Ensuring at least 2 to 3 cm of intra-abdominal esophagus is essential, as inadequate length predisposes to recurrence and persistent reflux [18]. The absence of tension at the GEJ confirms the adequacy of mobilization.

The hiatal defect is then closed by approximating the right and left crura with interrupted, non-absorbable sutures. This restores diaphragmatic continuity while maintaining flexibility at the hiatus. Mesh reinforcement is typically avoided to prevent complications such as erosion, fibrosis, or chronic pain [19]. The closure is calibrated over a 52 to 56 French bougie to prevent excessive narrowing and postoperative dysphagia.

Once the hiatus is secured, the gastric fundus is gently mobilized and anchored to the right crus and anterior crura using non-absorbable sutures. This fixation recreates the angle of His, an anatomic feature critical for the flap-valve mechanism that prevents reflux [20]. The anterior esophagus and esophago-phrenic ligament are subsequently anchored to the crura, reinforcing the GEJ's position and preventing cephalad migration. Together, these steps restore the hiatus's structural and functional integrity without altering the esophagus's circumference [21].

The procedure concludes with controlled desufflation of the pneumoperitoneum under direct visualization to confirm that all fixation points remain intact. Port sites are closed in the standard layered fashion. Patients are typically monitored for two to three days, with early ambulation and advancement of oral intake as tolerated. Early mobilization and enteral intake promote recovery, reduce pulmonary complications, and help confirm the restored physiological function of the GEJ [16].

Outcomes and efficacy

Clinical evidence on the BICORN procedure consistently demonstrates high patient satisfaction, durable symptom relief, and preservation of physiological function. Early data from European centers, particularly in Germany and Switzerland, report favorable outcomes with minimal adverse effects. Oelschlager et al. documented that approximately 91% of patients rated their postoperative results as good to very good, and more than 90% were able to discontinue proton pump inhibitors within months of surgery [17]. These findings underscore the effectiveness of BICORN in alleviating reflux symptoms while maintaining normal gastric physiology.

Unlike traditional wrap-based approaches, the BICORN technique achieves reflux control primarily through anatomical restoration rather than increased LES pressure. By repositioning the GEJ and reconstructing the angle of His, BICORN restores the natural antireflux barrier without altering the mechanics of swallowing or gastric venting. This anatomic conservation translates to a markedly lower incidence of dysphagia, gas-bloat syndrome, and inability to belch or vomit, complications that are common following fundoplication [18,19].

Postoperative physiologic assessments reveal that while LES pressures and transient relaxations remain near preoperative levels, most patients experience significant symptom resolution and improved quality of life [20]. This supports the principle that restoring anatomical alignment and hiatal competence may be sufficient for reflux control in appropriately selected patients, even without sphincter augmentation.

Long-term outcomes show that BICORN maintains durable symptom relief for most patients, though hernia recurrence remains a consideration. Reported recurrence rates range from 10% to 30% over 5 to 10 years, influenced by hernia size, suture technique, and patient-specific factors such as tissue quality and intra-abdominal pressure [17,21]. Studies of general laparoscopic hiatal hernia repair have documented similar recurrence rates, with most reoperations occurring within the first postoperative year [22]. Selectively using biological or absorbable mesh for large or recurrent hernias may reduce short-term recurrence rates to as low as 3% to 9%. However, long-term durability remains variable [23]. Importantly, mesh-related dysphagia occurs infrequently when the mesh does not circumferentially encircle the esophagus, emphasizing the importance of technical precision [24].

Overall, the BICORN procedure demonstrates high early and midterm efficacy, low complication rates, and strong patient-reported satisfaction. Its conservative, anatomy-preserving philosophy offers a compelling alternative to traditional antireflux surgeries, particularly for patients seeking physiologic restoration without the side effects of fundoplication. Long-term success depends on appropriate patient selection, meticulous surgical technique, and structured postoperative follow-up.

Complications and risk management

The BICORN procedure demonstrates a favorable safety profile and a low incidence of procedure-specific complications. By preserving the native gastroesophageal anatomy and avoiding a restrictive wrap, the procedure minimizes common postoperative adverse effects such as dysphagia, gas-bloat, and impaired gastric emptying, which are often seen in traditional antireflux surgeries [25-27].

Durable anatomical correction is achieved by ensuring adequate intra-abdominal esophageal length of at least 2 to 3 cm and repairing the hiatal defect with tension-balanced non-absorbable sutures. These measures reduce the risk of early recurrence and contribute to long-term stability of the GEJ [28,29]. Reported hernia recurrence rates after BICORN range from 10% to 30% over 5 to 10 years, influenced by hernia size, tissue quality, and surgical technique [30-33]. Recurrence is typically detected through follow-up imaging or symptomatic evaluation, with reoperation required in a minority of cases.

Physiological preservation is a key aspect of risk management in BICORN. By maintaining the natural angle of His and anchoring the stomach and esophagus without altering esophageal circumference, patients retain the ability to belch and vomit, enhancing postoperative quality of life and reducing functional complications [7]. Careful surgical technique, including precise mobilization of the esophagus and meticulous hiatal closure, is critical to minimizing tension on sutures and preventing herniation or suture-related complications [11].

Overall, the BICORN technique offers a balanced approach that combines effective reflux control with a low complication burden. Ongoing follow-up studies are needed to define further long-term outcomes, recurrence rates, and potential late complications. However, current evidence supports its role as a safe and physiologically conservative option for patients with hiatal hernia and GERD.

Future directions

While early results for the BICORN procedure are encouraging, several areas warrant further investigation to fully establish its role in managing hiatal hernia and GERD. Long-term prospective studies with larger patient cohorts are needed to assess durability, recurrence rates, and sustained symptom control beyond 5- to 10-year follow-up periods. These studies will help clarify how BICORN compares with established fundoplication techniques in terms of anatomical integrity and quality of life.

Randomized controlled trials directly comparing BICORN to Nissen and partial fundoplications are essential to rigorously evaluate differences in postoperative complications such as dysphagia, gas-bloat syndrome, and the ability to belch or vomit. Investigating patient selection criteria, particularly the impact of esophageal motility disorders, hernia size, and reflux severity, will optimize surgical outcomes by identifying patients who benefit most from BICORN versus traditional approaches.

Further research into biomechanical aspects of restoring the angle of His and techniques for hiatal closure could refine the procedure and reduce recurrence rates. Incorporating patient-reported outcome measures (PROMs) and cost-effectiveness analyses will also be important in understanding the broader clinical and economic impact of adopting BICORN in routine surgical practice.

## Conclusions

The BICORN procedure significantly advances the surgical treatment of GERD and hiatal hernia, prioritizing anatomical preservation and physiological function. Current evidence shows that BICORN provides effective symptom relief in over 90% of patients, with significantly lower rates of postoperative dysphagia and gas-bloat syndrome compared to traditional fundoplication. By restoring the angle of His and repairing the hiatal defect without constrictive wraps or mesh, BICORN offers a balanced approach that minimizes complications while maintaining durable anatomical correction. Although recurrence rates between 10% and 30% warrant continued vigilance, these outcomes are comparable to existing repair techniques. Importantly, BICORN fills a clinical niche for patients with mild to moderate reflux and preserved esophageal motility who may be unsuitable for or wish to avoid the functional impairments associated with fundoplication. To fully establish its role, well-designed randomized controlled trials and extended longitudinal studies are essential to evaluate long-term reflux control, recurrence, and quality-of-life outcomes. Refining patient selection criteria and surgical technique through ongoing research will maximize the benefits of this promising procedure. Overall, BICORN offers a valuable addition to the antireflux surgical options, aligning surgical success with improved postoperative quality of life.
